# Supplementary material for: Visualization and molecular characterization of whole-brain vascular networks with capillary resolution
Source: Nat Commun. 2020 Feb 27;11:1104. doi: 10.1038/s41467-020-14786-z (PMC7046771; doi:10.1038/s41467-020-14786-z)
Supplement: Supplementary file 1 — Supplementary Information [file 41467_2020_14786_MOESM1_ESM.pdf]

Supplementary Information

Visualization and molecular characterization of whole-brain vascular networks with capillary resolution

Miyawaki *et al.*

SUPPLEMENTARY FIGURES

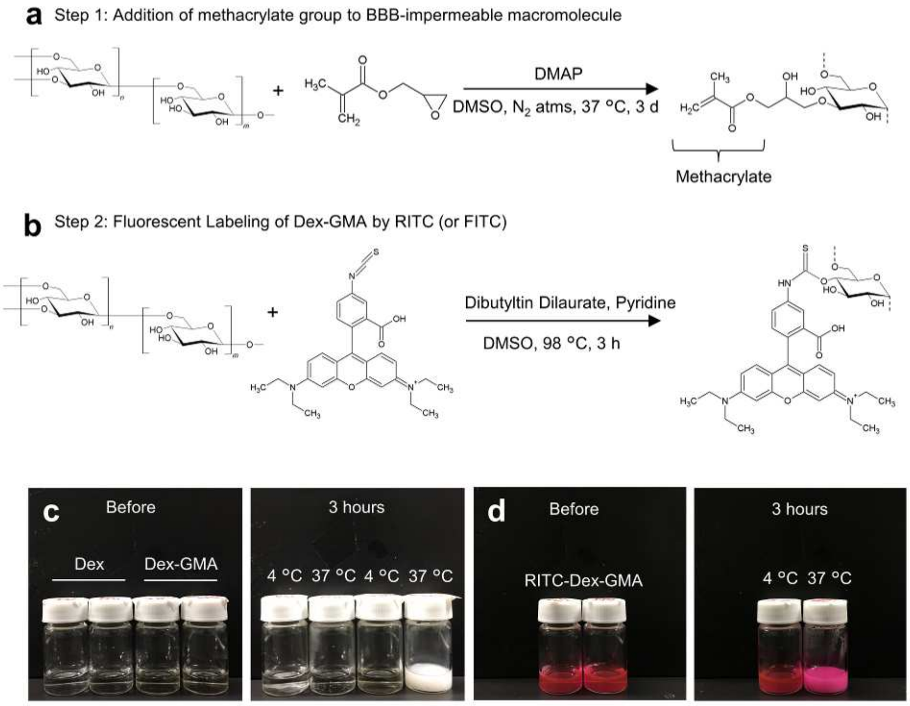

Supplementary Figure 1. Synthesis of fluorescent crosslinker and thermally controlled formation of the Dex-GMA-acrylamide hydrogel

(a, b) The reactions for the synthesis of the BBB-impermeable fluorescent crosslinker RITC-Dex-GMA. First, glycidyl methacrylate is conjugated to dextran under a nitrogen atmosphere. Then, RITC is conjugated to Dex-GMA. FITC can be conjugated by the same reaction.

(c) Mixture of 5% dextran or Dex-GMA, 4% acrylamide, and 0.25% VA-044 before and after 3-h incubation at 4 or 37°C. In the presence of Dex-GMA, white hydrogels formed in a temperature-dependent manner.

(d) Mixture of 5% RITC-Dex-GMA, 4% acrylamide, and 0.25% VA-044 before and after 3-h incubation at 4 or 37°C. Red-fluorescent hydrogels formed at 37°C.

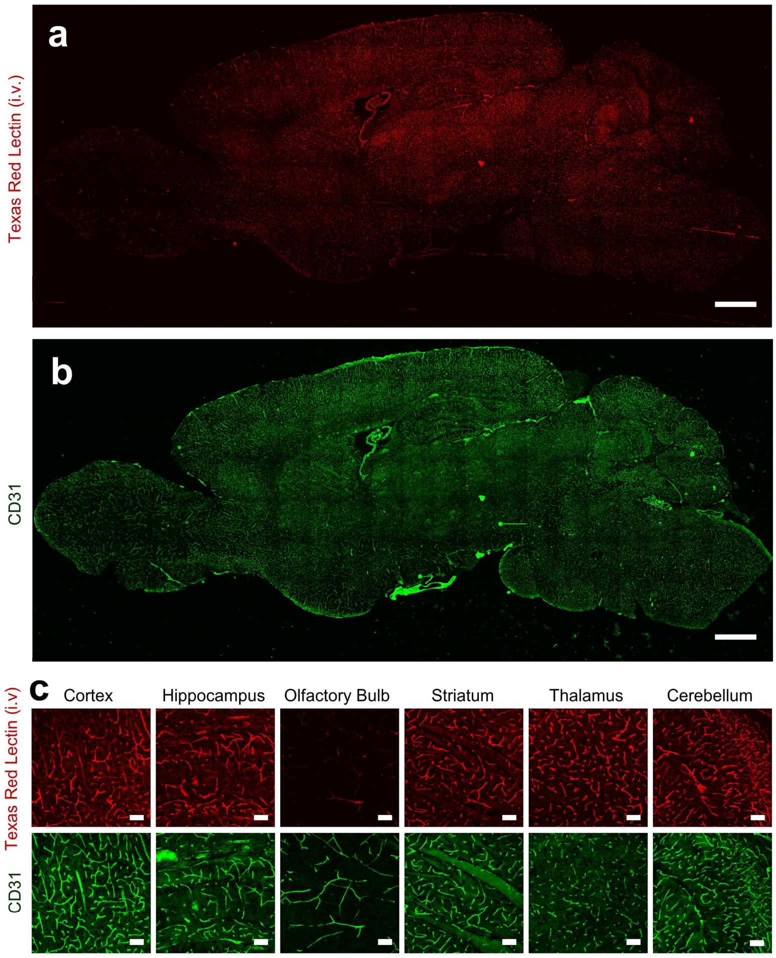

Supplementary Figure 2. Staining of cerebral vasculature by anti-CD31 antibody and intravenous injection of lectin-conjugated dye

(a, b) Confocal images of endothelial cells labeled with intravenous injection of Texas Red-conjugated lectin (a, red) and immunohistochemistry against CD31 (b, green). Scale bar = 1 mm.

(c) Magnified images of (a) and (b) at different brain regions. Scale bar = 100 μm.

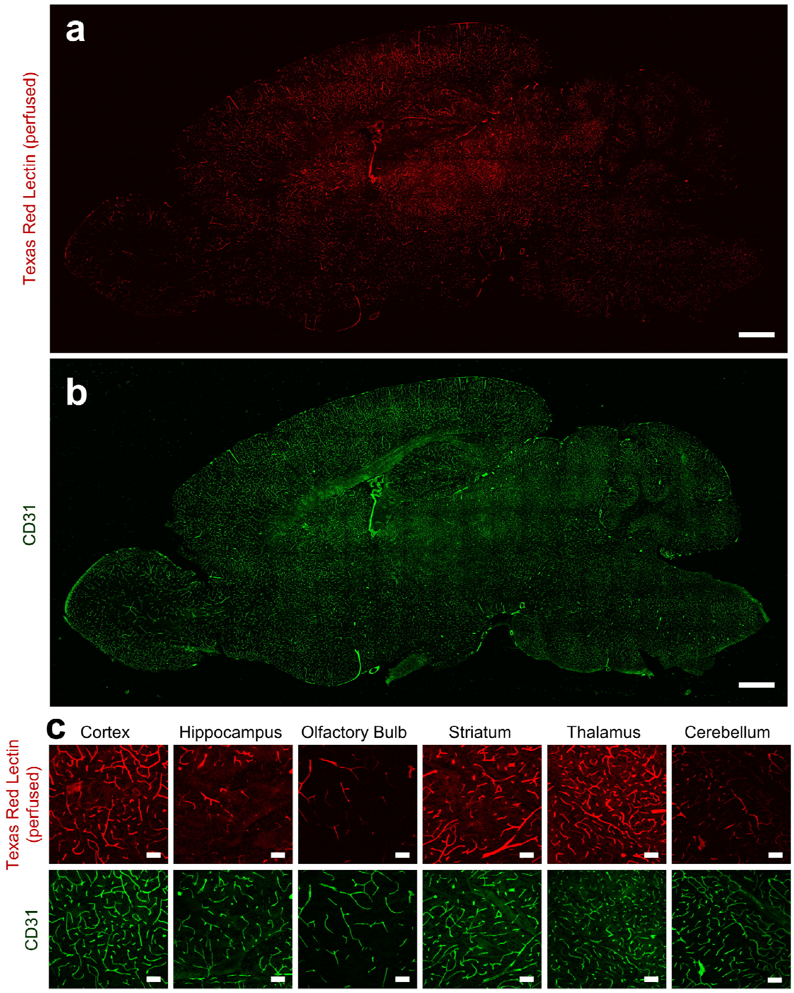

Supplementary Figure 3. Staining of cerebral vasculature by anti-CD31 antibody and transcardially perfused lectin-conjugated dye

(a, b) Confocal images of endothelial cells labeled by transcardial perfusion of Texas Red-conjugated lectin (a, red) and endothelial cells immunostained against CD31 (b, green). Scale bar = 1 mm.

(c) Magnified images of (a) and (b) in different brain regions. Scale bar = 100 μm.

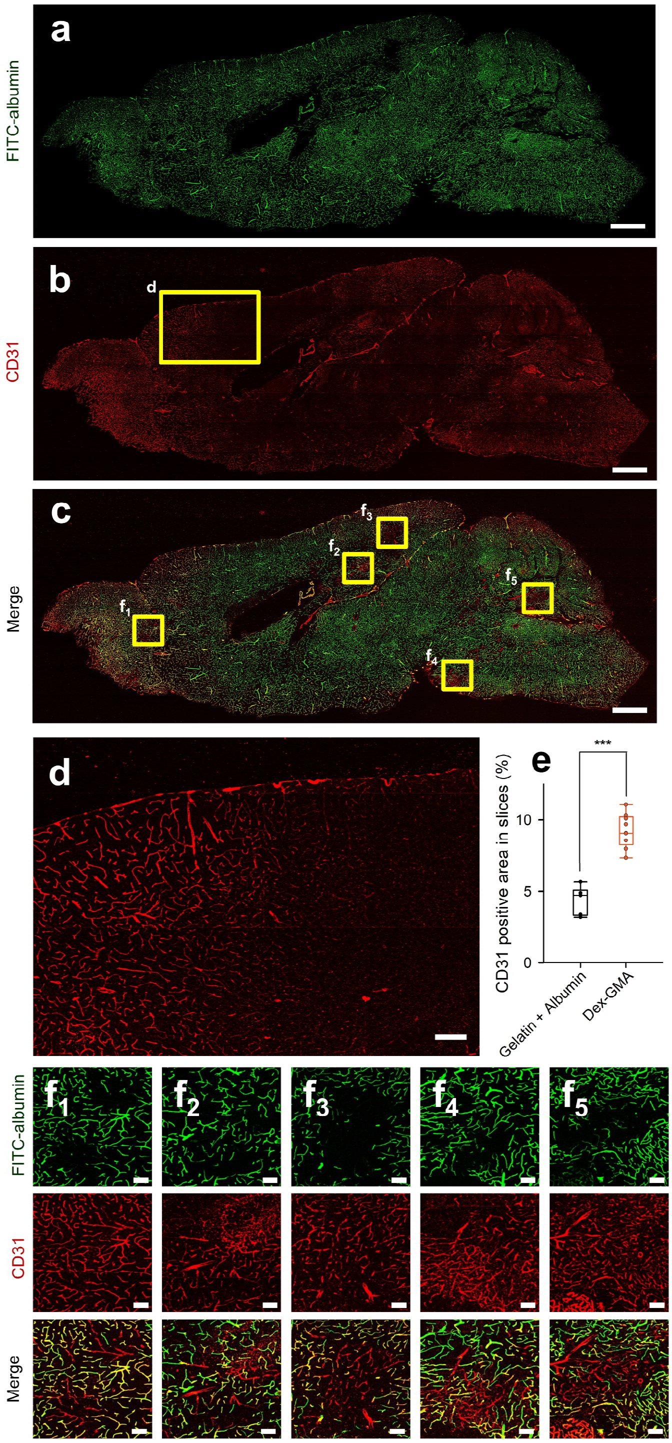

Supplementary Figure 4. Staining of cerebral vasculature by gelatin-FITC-albumin gel and anti-CD31

(a-c) Confocal images of endothelial cells labeled with perfusion of gelatin-FITC-albumin gel (a, green), immunohistochemistry against CD31 (b, red) and their merged image (c). Scale bar = 1 mm.

(d) Magnified image of a boxed region in (b). Scale bar = 200 μm.

(e) The percentages of CD31 immuno-positive areas in the slices prepared from gelatin-FITC-albumin- or RITC-Dex-GMA-treated samples (n = 5 and 9 slices from 5 or 9 mice, respectively. Box plots indicate the medians and 25−75% interquartile ranges; whiskers cover 10−90% quantiles. ***$P = 5.83 \times 10^{-6}$, $t_{12} = 7.66$, Student's t-test).

(f) Examples of brain regions without FITC signal. Images are magnified from (a-c). Scale bar = 100 μm. Data are provided in the Source Data file.

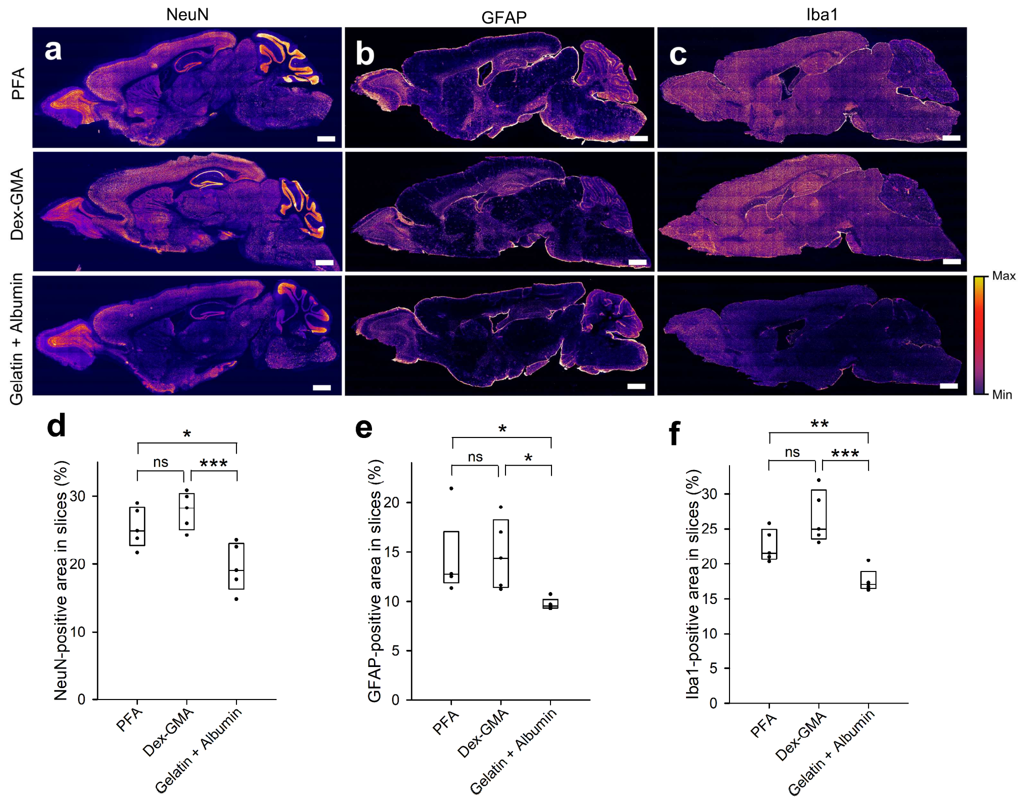

Supplementary Figure 5. Comparison of antigenicity in PFA-treated, Dex-GMA-treated, and gelatin-albumin gel-treated samples

(a-c) Slices prepared from PFA-treated (top), Dex-GMA-treated (middle), or gelatin-albumin treated samples (bottom) were immunostained against NeuN (a), GFAP (b) or Iba1 (c). The conditions for staining, image acquisition, and image processing were fixed within the same antibody. Scale Bar = 1 mm.

(d-f) The percentages of NeuN (d), GFAP (e), or Iba1 (f) -immunopositive areas in the whole slices were compared among PFA-treated, Dex-GMA-treated, and gelatin-albumin treated samples (n = 5 slices from 5 mice each. Box plots indicate the medians and 25−75% interquartile ranges. NeuN: $P = 3.47 \times 10^{-3}$, $F_{2,12} = 9.44$, one-way ANOVA; PFA *versus* Dex-GMA: $P = 0.461$, $Q_{2,12} = 1.734$; Dex-GMA *versus* gelatin-albumin: ***$P = 3.13 \times 10^{-3}$, $Q_{2,12} = 5.972$; PFA *versus* gelatin-albumin: *$P = 2.79 \times 10^{-2}$, $Q_{2,12} = 4.238$, Tukey's test. GFAP: $P = 9.19 \times 10^{-3}$, $H_{2,12} = 9.38$, Kruskal-Wallis test; PFA *versus* Dex-GMA: $P = 0.10$, $Q_{2,12} = 0.99$; Dex-GMA *versus* gelatin-albumin: *$P = 2.45 \times 10^{-2}$, $Q_{2,12} = 2.61$; PFA versus gelatin-albumin: *$P = 2.45 \times 10^{-2}$, $Q_{2,12} = 2.61$, Steel-Dwass test. Iba1: $P = 7.60 \times 10^{-4}$, $F_{2,12} = 13.9$, one-way ANOVA; PFA *versus* Dex-GMA: $P = 8.48 \times 10^{-2}$, $Q_{2,12} = 3.342$; Dex-GMA *versus* gelatin-albumin: ***$P = 5.48 \times 10^{-4}$, $Q_{2,12} = 7.434$; PFA *versus* gelatin-albumin: **$P = 3.35 \times 10^{-2}$, $Q_{2,12} = 4.092$, Tukey's test). Data are provided in the Source Data file.

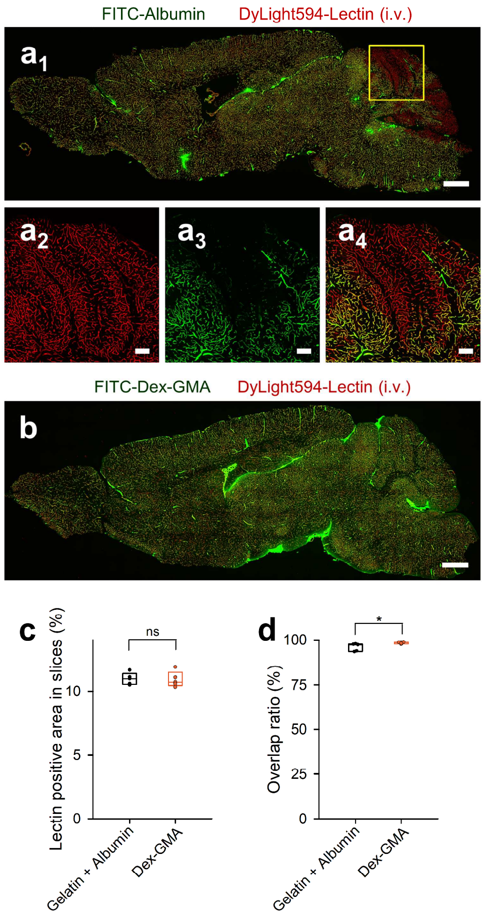

Supplementary Figure 6. Comparison of staining performances between gelatin-FITC-albumin and FITC-Dex-GMA

(a) Confocal images of a sagittal brain slice prepared from an adult mouse treated with intravenous injection of DyLight594-conjugated lectin (red) and perfusion of gelatin-FITC-albumin gel (green). ($a_{2\text{-}4}$) Examples of brain regions without FITC signal. Images are magnified from (a_1). Scale bar = 1 mm (a_1) and 200 μm ($a_{2\text{-}4}$).

(b) The same as panel (a_1), but vasculature was labeled with intravenous injection of DyLight594-conjugated lectin (red) and perfusion of FITC-Dex-GMA (green). Scale bar = 1 mm.

(c) The percentages of lectin-positive areas in the slices prepared from gelatin-FITC-albumin- or FITC-Dex-GMA-treated samples (n = 5 slices from 5 mice each. Box plots indicate the medians and 25−75% interquartile ranges, P = 0.892, t_8 = 0.14, Student's t-test).

(d) Overlap ratios between lectin-positive areas and FITC-positive areas in gelatin-FITC-albumin- or Dex-GMA-treated samples (n = 5 slices from 5 mice each. Box plots indicate the medians and 25−75% interquartile ranges, *P = 4.72 × 10^{-2}, t_8 = 2.34, Student's t-test). Data are provided in the Source Data file.

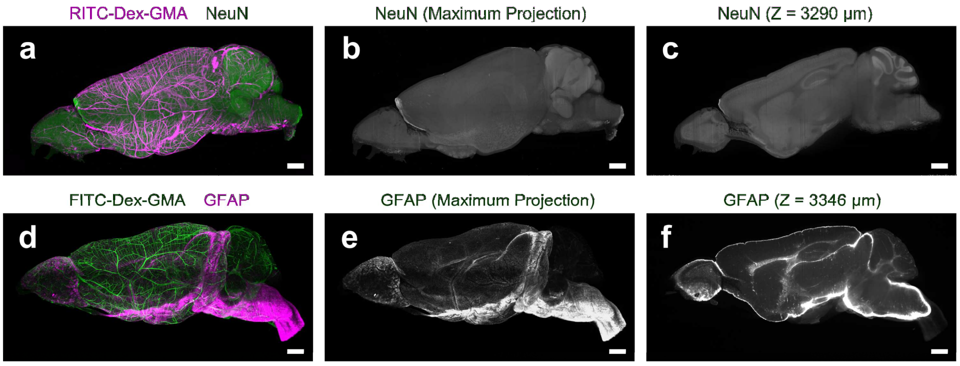

Supplementary Figure 7. SeeNet is compatible with other antibodies

(a-c) Maximum image projections of cerebral hemispheres. The vasculature was cast using RITC-Dex-GMA (a, magenta). After delipidation with SDC, the hemisphere was immunostained against NeuN (green in (a) and gray in (b)). (c) An optical section of (b) at $Z = 3290$ μm. Scale bar = 1 mm.

(d-f) The same as panels (a-c), but the vasculature was cast using FITC-Dex-GMA (d, green) and immunostained against GFAP (magenta in (d) and gray in (e)). (f) The optical section was taken at $Z = 3346$ μm. Scale bar = 1 mm.

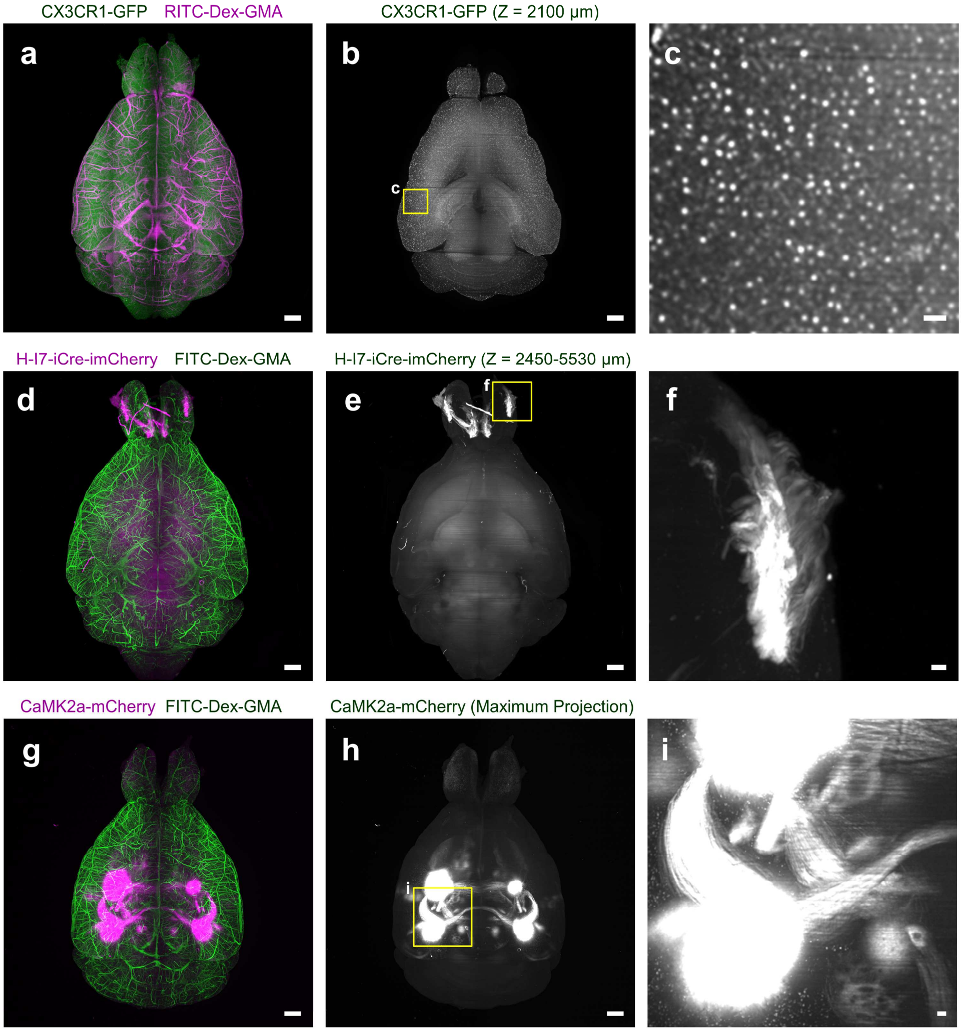

Supplementary Figure 8. SeeNet is compatible with other fluorescent proteins

(a) Maximum projection of a SeeNet (RITC-Dex-GMA)-treated brain of a CX3CR1-GFP transgenic mouse. Scale bar = 1 mm.

(b) The GFP signal in (a) was optically sectioned at *Z* = 2100 μm. Scale bar = 1 mm.

(c) The boxed region in (b) is magnified. Scale bar = 100 μm.

(d) Maximum image projection of a SeeNet (FITC-Dex-GMA)-treated brain of a H-I7-iCre-imCherry mouse. Scale bar = 1 mm.

(e) Maximum projection of the mCherry signal between *Z* = 2450-5530 μm. Scale bar = 1 mm.

(f) The boxed region in (e) is magnified, indicating that axons from olfactory sensory neurons are segregated into glomeruli. Scale bar = 100 μm.

(g-i) The same as (d-f) but for a brain that received bilateral injections of AAVdj-CaMKIIa-mCherry into the S1 and V1 neocortex 2 w prior to sampling.

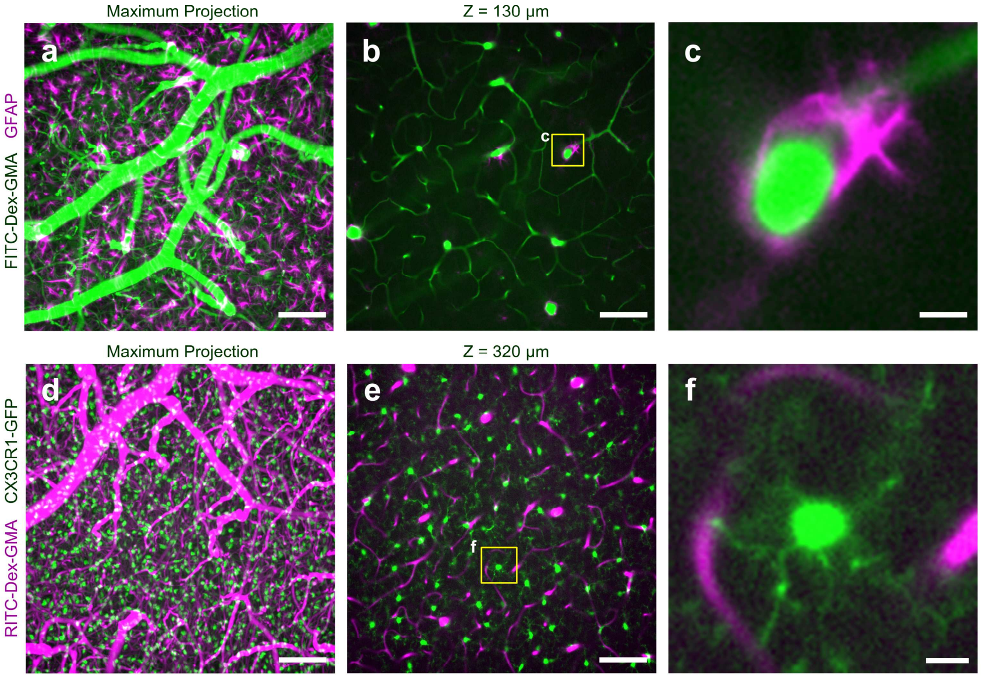

Supplementary Figure 9. Observations of glial microstructures and vessels

(a) Maximum image projection of a GFAP-immunostained (magenta), SeeNet-treated (green) brain.

(b) The sample of (a) was optically sectioned at Z = 130 μm. Scale bar = 100 μm.

(c) The boxed region in (b) is magnified. Scale bar = 10 μm.

(d) Maximum image projection of a SeeNet-treated (magenta) brain of a CX3CR1-GFP (green) mouse.

(e) The sample of (d) was optically sectioned at Z = 320 μm. Scale Bar = 100 μm.

(f) The boxed region in (e) is magnified. Scale bar = 10 μm.

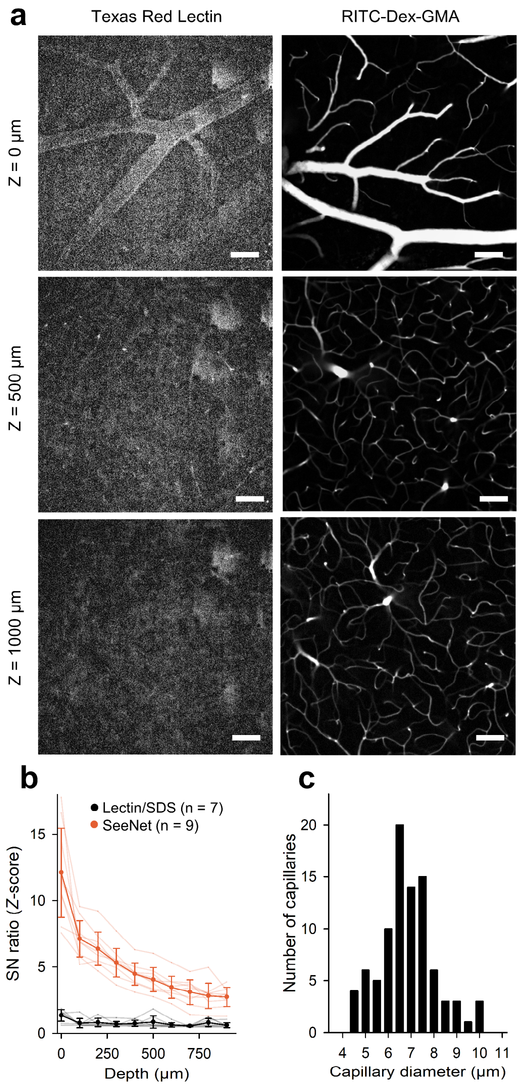

Supplementary Figure 10. SeeNet-treated samples have high SN ratios in 3D imaging

(a) Comparisons of volumetric imaging data obtained from a sample prepared with a conventional protocol (left: intravenous injection of Texas Red-conjugated lectin, delipidation by SDS, and refractive index matched by Sca*l*eCUBIC-2) or SeeNet (right). Scale bar = 100 µm.

(b) The SN ratios of confocal images of the cleared brain were plotted *versus* depth from the pia (10×, numerical aperture = 0.4, working distance = 2.17 mm; *n* = 7 and 9 mice, respectively. $P = 2.22 \times 10^{-16}$, $F_{1,140} = 5.55 \times 10^{2}$, two-way ANOVA). Error bars represent standard deviations.

(c) Distribution of the diameters of capillaries in SeeNet-treated samples. The mean ± SD was 6.58 ± 1.21 µm (*n* = 90 capillaries from 9 mice). Data are provided in the Source Data file.

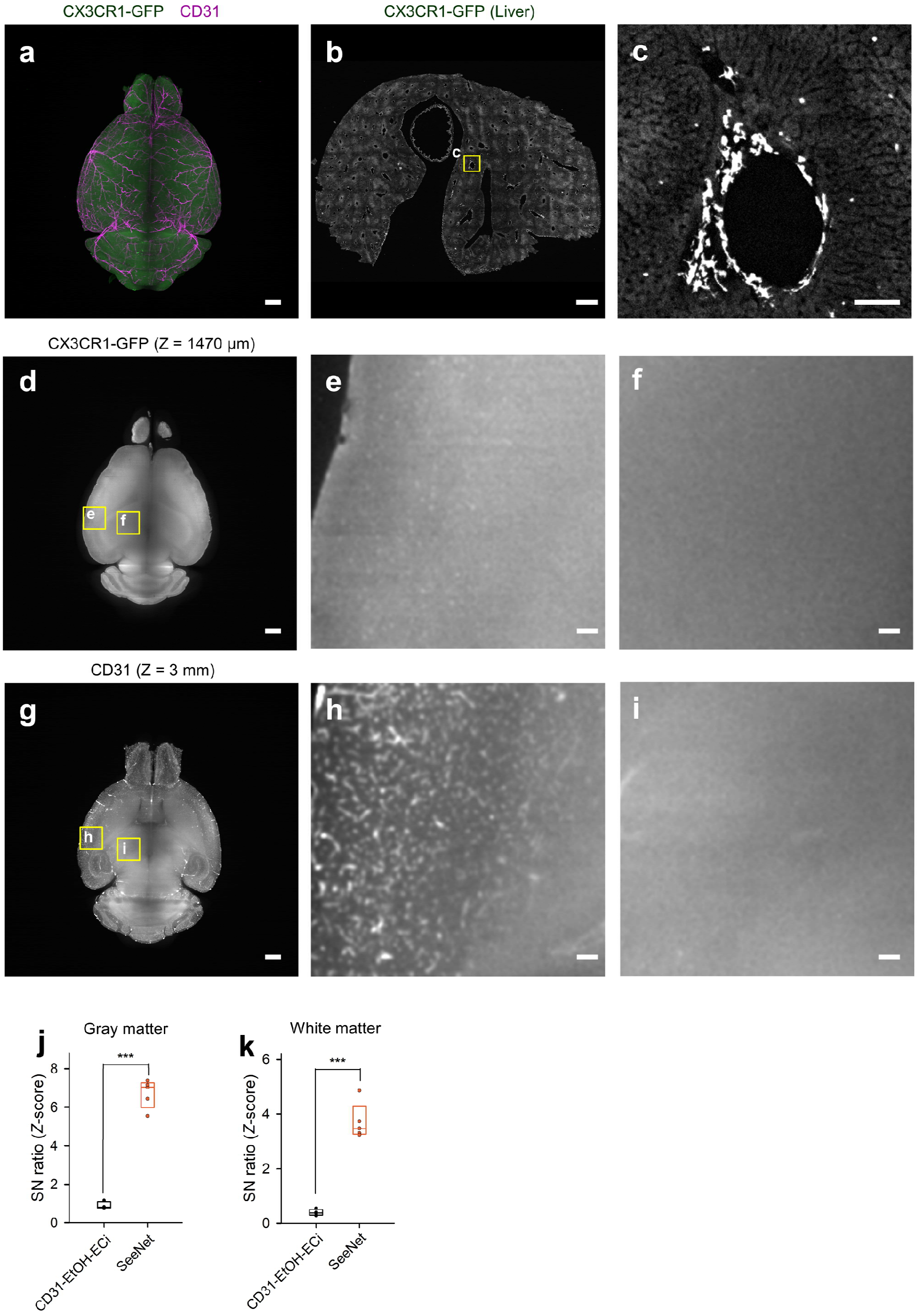

Supplementary Figure 11. 3D visualization of cerebral vasculature by anti-CD31 and EtOH-ECi

(a) Maximum image projection of a brain of a CX3CR1-GFP transgenic mouse that received intravenous injection of anti-CD31-Alexa 647 and was cleared by EtOH-ECi. Scale bar = 1 mm.

(b, c) Confocal images of CX3CR1-GFP signals in the liver (uncleared) of the same

mouse used in (a). The boxed region in (b) is shown in (c). Scale bar = 1 mm and 100 μm each.

(d) The GFP signal in (a), shown on a grayscale, was optically sectioned at Z = 1470 μm. Scale bar = 1 mm.

(e, f) Two boxed regions in (d) are magnified in (e) and (f). Scale bar = 100 μm. Also see Supplementary Figure 8c for comparison with SeeNet-treated brains.

(g) The CD31 signal in (a), shown on a gray scale, was optically sectioned at Z = 3 mm. Scale bar = 1 mm.

(h, i) Two boxed regions in (g) are magnified. Scale bar = 100 μm. Also see Figure 5b-d for comparison with SeeNet-treated brains.

(j, k) SN ratios of the fluorescence intensities in the gray matter (j) and white matter (k) of the CD31-EtOH-ECi treated samples and SeeNet-treated samples (n = 4 and 5 optical slices from 4 or 5 mice, respectively; box plots indicate the medians and 25−75% interquartile ranges. j; ***$P = 1.32 \times 10^{-6}$, $t_7 = 15.1$, k; **$P = 2.53 \times 10^{-5}$, $t_7 = 9.75$, Student's t-test). Data are provided in the Source Data file.

SUPPLEMENTARY TABLES

	S/N	False Negative	Need for Tg mice	Antigency	Tissue clearing compatibiltiy	Approx cost (USD/sample)	Reference
Texas Red Lectin (tail vein)	Low	Lumens not filled (not suitable for vectorization)	No	Preserved	Severe loss of signal	20	This paper
Texas Red Lectin (perfusion with PBS)	Medium	Uneven labeling (Venules not stained)	No	Preserved	Severe loss of signal	20	This paper
CD31 Antibody (tail vein)	Low	Lumens not filled	No	Preserved	Severe loss of signal	20	Qi et al., 2019[1]
Tie2-GFP	High	Uneven labeling, lumens not filled	Single Tg	Preserved	Yes	The initial + maintenance cost	Motoike et al., 2000[2]
Tie2-Cre and Reporter line	High	Lumens not filled	Double Tg	Preserved	Yes	The initial + maintenance cost	Kisanuki et al., 2001[3], Jing et al., 2018[4]
Gelatin & FITC-albumin	High	Prone to be clogged	No	Partially lost	Yes	200	Tsai et al., 2009[5], Di Giovanna, 2018[6]
Vascular Corrosion Casting	High	Prone to be clogged	No	Lost	No	4	Walker et al., 2011[7]
Hybrid Hydrogel	High	Near-Complete	No	Preserved	Yes	10 (FITC), 20 (RITC)	This paper

Supplementary Table 1. Methods for visualization of the vasculature

		Change in volume	Molecular information	Special setup	Time taken to prepare specimen	Reference
Vascular Corrosion Casting		Slight Shrinking	Lost	No	10 days	Walker et al., 2011[7]
Serial Sectioning*		No	Preserved (but difficlut to be coupled with IHC)	Yes	0	Xue et al., 2014[8]
Tissue Clearing**	CLARITY (ETC)	Slight Swelling	Preserved	Yes	10 days (difficult to be parallelized)	Chung et al., 2013[9]
	CLARITY (passive)	Slight Swelling	Preserved	No	6-8 weeks	Chung et al., 2013[9]
	PACT	Heavily Swelling	Preserved	No	2 weeks	Yang et al., 2014[10]
	CUBIC L/R	Heavily Swelling	Preserved	No	1 week	Kubota et al., 2017[11]
	iDISCO	Shrinking	Partially preserved	No	1 week (+ immunostaining)	Renier et al., 2014[12]
	uDISCO	Shrinking	Partially preserved	No	1 week	Pan et al., 2016[13]
	FDISCO	Shrinking	Preserved	No	4 Days	Qi et al., 2019[1]
	SDC-CUBIC2	Slight Swelling	Preserved	No	10 days	This Paper

Supplementary Table 2. Methods for 3D observation

*Complete alignment of all images through a large sample (ex. mouse brain) is still challenging.

**Other clearing protocols are not shown in this table because their optical clearing potency was not strong enough to achieve whole-brain imaging.

SUPPLYMENTARY REFERENCES

1. Qi, Y., et al. FDISCO: Advanced solvent-based clearing method for imaging whole organs. *Science advances*, **5**, eaau8335. (2019).
2. Motoike, T., et al. Universal GFP reporter for the study of vascular development. *Genesis* **28**, 75-81 (2000).
3. Kisanuki, Y.Y., et al. Tie2-Cre transgenic mice: a new model for endothelial cell-lineage analysis in vivo. *Dev Biol* **230**, 230-242 (2001).
4. Jing, D., et al. Tissue clearing of both hard and soft tissue organs with the PEGASOS method. *Cell Res* **28**, 803-818 (2018).
5. Tsai, P.S., et al. Correlations of neuronal and microvascular densities in murine cortex revealed by direct counting and colocalization of nuclei and vessels. *J Neurosci* **29**, 14553-14570 (2009).
6. Di Giovanna, A.P., et al. Whole-Brain Vasculature Reconstruction at the Single Capillary Level. *Scientific reports* **8**, 12573 (2018).
7. Walker, E.J., Shen, F., Young, W.L. & Su, H. Cerebrovascular casting of the adult mouse for 3D imaging and morphological analysis. *J Vis Exp*, e2958 (2011).
8. Xue, S., et al. Indian-ink perfusion based method for reconstructing continuous vascular networks in whole mouse brain. *PLoS One* **9**, e88067 (2014).
9. Chung, K., et al. Structural and molecular interrogation of intact biological systems. *Nature* **497**, 332-337 (2013).
10. Yang, B., et al. Single-cell phenotyping within transparent intact tissue through whole-body clearing. *Cell* **158**, 945-958 (2014).
11. Kubota, S.I., et al. Whole-Body Profiling of Cancer Metastasis with Single-Cell Resolution. *Cell reports* **20**, 236-250 (2017).
12. Renier, N., et al. iDISCO: a simple, rapid method to immunolabel large tissue samples for volume imaging. *Cell* **159**, 896-910 (2014).
13. Pan, C., et al. Shrinkage-mediated imaging of entire organs and organisms using uDISCO. *Nat Methods* **13**, 859-867 (2016).
