# Supplementary material for: Visualization and molecular characterization of whole-brain vascular networks with capillary resolution
Source: Nat Commun. 2020 Feb 27;11:1104. doi: 10.1038/s41467-020-14786-z (PMC7046771; doi:10.1038/s41467-020-14786-z)
Supplement: Supplementary file 3 — Description of Additional Supplementary Files [file 41467_2020_14786_MOESM3_ESM.pdf]

**Description of Additional Supplementary Files**

**Supplementary Movie 1. Whole-brain imaging of a SeeNet-treated sample using light-sheet fluorescence microscopy**

**Supplementary Movie 2. Single-capillary tracing from the whole-brain imaging data**

**Supplementary Movie 3. Cortico-hippocampal vascular connections of the dorsoposterior hemisphere in whole-brain imaging data**

The RITC-Dex-GMA signal in the posterior part of the left hemisphere is shown in gray. A vascular path connecting the cortical penetrating vessel (the thick and bright vessel running vertically to the pia) and the hippocampal transverse vessel (the thick vessel running horizontally) is traced in magenta.

**Supplementary Movie 4. Arterioles of the dorsoposterior hemisphere in whole-brain imaging data**

The same video as Supplementary Movie 3 but showing the anti-αSMA signal in gray. Traces of cortico-hippocampal vascular paths observed in Supplementary Movie 3 are highlighted in magenta, cyan or yellow. The vascular paths were identified as arteriole-arteriole (magenta), venule-arteriole (cyan), or venule-venule (yellow) connections.

**Supplementary Movie 5. Volumetric imaging of the cortical and hippocampal vasculature**

The neocortex located above the hippocampus and dorsal hippocampus in a SeeNet-treated brain was volumetrically imaged by confocal microscopy. The RITC-Dex-GMA signal is shown in gray. A vascular path connecting a cortical penetrating vessel and a hippocampal transverse vessel is traced in magenta.

**Supplementary Movie 6. Volumetric imaging of the cortical and hippocampal arteriole**

The same video as Supplementary Movie 5 but showing the anti-αSMA signal in gray. The cortical penetrating vessel and the hippocampal transverse vessel were both α-SMA immunopositive.
