# Supplementary material for: Visualization and molecular characterization of whole-brain vascular networks with capillary resolution
Source: Nat Commun. 2020 Feb 27;11:1104. doi: 10.1038/s41467-020-14786-z (PMC7046771; doi:10.1038/s41467-020-14786-z)
Supplement: Supplementary file 10 — Reporting Summary [file 41467_2020_14786_MOESM10_ESM.pdf]

nature research

Corresponding author(s): Takeyuki Miyawaki

Last updated by author(s): Jan 1, 2020

Reporting Summary

Nature Research wishes to improve the reproducibility of the work that we publish. This form provides structure for consistency and transparency in reporting. For further information on Nature Research policies, see Authors & Referees and the Editorial Policy Checklist.

Statistics

For all statistical analyses, confirm that the following items are present in the figure legend, table legend, main text, or Methods section.

n/a	Confirmed	
☐	☒	The exact sample size (n) for each experimental group/condition, given as a discrete number and unit of measurement
☐	☒	A statement on whether measurements were taken from distinct samples or whether the same sample was measured repeatedly
☐	☒	The statistical test(s) used AND whether they are one- or two-sided<br>*Only common tests should be described solely by name; describe more complex techniques in the Methods section.*
☐	☒	A description of all covariates tested
☐	☒	A description of any assumptions or corrections, such as tests of normality and adjustment for multiple comparisons
☐	☒	A full description of the statistical parameters including central tendency (e.g. means) or other basic estimates (e.g. regression coefficient) AND variation (e.g. standard deviation) or associated estimates of uncertainty (e.g. confidence intervals)
☐	☒	For null hypothesis testing, the test statistic (e.g. F, t, r) with confidence intervals, effect sizes, degrees of freedom and P value noted<br>*Give P values as exact values whenever suitable.*
☒	☐	For Bayesian analysis, information on the choice of priors and Markov chain Monte Carlo settings
☒	☐	For hierarchical and complex designs, identification of the appropriate level for tests and full reporting of outcomes
☒	☐	Estimates of effect sizes (e.g. Cohen's d, Pearson's r), indicating how they were calculated

Our web collection on statistics for biologists contains articles on many of the points above.

Software and code

Policy information about availability of computer code

Data collection	For whole brain imaging by the light sheet microscopy, confocal imaging by CV1000 and light transmittance measurement by Spectral Haze Meter SH 7000, binary codes provided by manufacturer were used.
Data analysis	MATLAB, Image J and R were used. We used custom scripts for the analysis. The codes are available from the corresponding author upon reasonable request.

For manuscripts utilizing custom algorithms or software that are central to the research but not yet described in published literature, software must be made available to editors/reviewers. We strongly encourage code deposition in a community repository (e.g. GitHub). See the Nature Research guidelines for submitting code & software for further information.

Data

Policy information about availability of data

All manuscripts must include a data availability statement. This statement should provide the following information, where applicable:

- Accession codes, unique identifiers, or web links for publicly available datasets
- A list of figures that have associated raw data
- A description of any restrictions on data availability

All the data that support the findings of this study are available from the corresponding author upon reasonable request.

Field-specific reporting

Please select the one below that is the best fit for your research. If you are not sure, read the appropriate sections before making your selection.

☒ Life sciences ☐ Behavioural & social sciences ☐ Ecological, evolutionary & environmental sciences

Life sciences study design

All studies must disclose on these points even when the disclosure is negative.

Sample size	The sample sizes were determined based on the literatures in the fields. No statistical tests were used to predetermine sample sizes. Sample sizes for all experiments are described as "n=??".
Data exclusions	No data were excluded from the analysis.
Replication	All experiments were replicated at least three times except those associated with Supplementary Figure 8, 9, 11d-f, which were not replicated due to limited availability of transgenic mice.
Randomization	The mice used in this study were randomly chosen from colonies. For the comparison of tissue-clearing ability of SDS and SDC (Supplementary figure 3), the same number of brains were treated with either detergents at the same time.
Blinding	No blinding was done in this study because knowledge of experimental conditions was required during data collection. The binarization of the images was conducted in automated matter as described in the Method section.

Reporting for specific materials, systems and methods

We require information from authors about some types of materials, experimental systems and methods used in many studies. Here, indicate whether each material, system or method listed is relevant to your study. If you are not sure if a list item applies to your research, read the appropriate section before selecting a response.

Materials & experimental systems

n/a	Involved in the study	
☐	☒	Antibodies
☒	☐	Eukaryotic cell lines
☒	☐	Palaeontology
☐	☒	Animals and other organisms
☒	☐	Human research participants
☒	☐	Clinical data

Methods

n/a	Involved in the study	
☒	☐	ChIP-seq
☒	☐	Flow cytometry
☒	☐	MRI-based neuroimaging

Antibodies

Antibodies used	Rat anti-CD31 (1:300; BD Biosciences, 550274, Franklin Lakes, NJ), Rabbit anti-Iba1 (1:200, Wako Pure Chemical Industries Ltd., 019-19741, Japan), Rabbit anti-NeuN (1:300; ABN-78, Sigma-Aldrich), Rabbit anti-GFAP (1:300; Z0334, Agilent Technologies, Santa Clara, CA), FITC-conjugated mouse anti-αSMA (1:100, Sigma Aldrich., F3777, St. Louis, MO), Alexa 488-conjugated mouse anti-NeuN (1:100; MAB377X, Merck-Millipore, Burlington, MA), Cy3-conjugated mouse anti-GFAP (1:100; C9205, Sigma-Aldrich), anti-CD31-Alexa 647 (102416; BioLegend, San Diego, CA), Alexa 488-labeled goat anti-rat IgG (1:300; Thermo Fisher Scientific, A-11006, Waltham, MA), Alexa 594-labeled goat anti-rat IgG (1:300; Thermo Fisher Scientific, A-11007, Waltham, MA), Alexa 594-labeled goat anti-rabbit IgG (1:300; Thermo Fisher Scientific, A-11012, Waltham, MA), and Alexa 594-labeled donkey anti-rabbit IgG (1:300; R-37119, Thermo Fisher Scientific).
Validation	Rat anti-CD31 was shown to stain the endothelial cells of zinc-fixed paraffin-embedded section of U-87 MG tumor in mouse brain by the manufacturer. Mouse anti-NeuN was shown to stain nucleus of the neurons in the granular layer of rat cerebellum by the manufacturer. Cy3-conjugated mouse anti-GFAP was shown to stain astrocytes and Bergman glia cells in tissue sections (Debus, E., 1983). Rabbit anti-GFAP was shown to stain astrocyte of the mouse brain sections by the manufacturer. Specificities of rabbit anti-Iba1, rabbit anti-NeuN, and FITC-conjugated mouse anti-αSMA were validated by the manufacturers using western blot assay. anti-CD31-Alexa 647 is quality control tested by immunofluorescent staining with flow cytometric analysis. Alexa 488/594-labeled goat anti-rat IgG, Alexa 594-labeled donkey anti-rabbit IgG, and Alexa 594-labeled goat anti-rabbit IgG were shown to immunohistochemically stain the corresponding primary antibodies by the manufacturer.

Animals and other organisms

Policy information about studies involving animals; ARRIVE guidelines recommended for reporting animal research

Laboratory animals	Two- to five-month-old C57BL/6J (Japan SLC, Inc., Japan), CX3CR1 –GFP (Stock No: 005582, The Jackson Laboratory), H-I7-iCre-imCherry (Accession No. CDB0537T, RIKEN LARGE), and Arc-dVenus transgenic mice were used in this study. All animals were housed under a 12:12-h dark–light cycle (light from 07:00 to 19:00) at 22 ± 1°C with ad libitum access to food and water.
Wild animals	n/a

Field-collected samples	n/a
Ethics oversight	Animal experiments were performed with the approval of the Animal Experiment Ethics Committee at the University of Tokyo (approval number: 24-70) and according to the University of Tokyo guidelines for the Care and Use of Laboratory Animals. All experimental protocols were carried out in accordance with the Fundamental Guidelines for Proper Conduct of Animal Experiment and Related Activities in Academic Research Institutions (Ministry of Education, Culture, Sports, Science and Technology, Notice No. 71 of 2006), the Standards for Breeding and Housing of and Pain Alleviation for Experimental Animals (Ministry of the Environment, Notice No. 88 of 2006) and the Guidelines on the Method of Animal Disposal (Prime Minister's Office, Notice No. 40 of 1995).

Note that full information on the approval of the study protocol must also be provided in the manuscript.
